# The Global NeuroSurg Research Collaborative: A Novel Student-Based Model to Expand Global Neurosurgery Research

**DOI:** 10.3389/fsurg.2021.721863

**Published:** 2021-10-27

**Authors:** Ahmed Negida, Ahmed M. Raslan

**Affiliations:** ^1^Global NeuroSurg Research Collaborative, Oregon Health and Science University, Portland, OR, United States; ^2^Global Neurosurgery Initiative, Program of Global Surgery and Social Change, Harvard Medical School, Boston, MA, United States; ^3^School of Pharmacy and Biomedical Sciences, University of Portsmouth, Portsmouth, United Kingdom; ^4^Faculty of Medicine, Zagazig University, Zagazig, Egypt; ^5^Neurological Surgery Department, Oregon Health and Science University, Portland, OR, United States

**Keywords:** global neurosurgery, neurosurgery—head trauma—chronic subdural hematoma—burr hole—craniotomy, medical students, research collaboration, low and middle income countries

The Global NeuroSurg research collaborative (www.globalneurosurg.org) was initiated in May 2018 to (1) study the existing variations in management and outcomes of neurosurgical conditions between low- and middle-income countries (LMICs) and high-income countries and (2) determine the practices and factors associated with the best neurosurgical outcomes, therefore, helping to improve the current neurosurgical care. The collaborative is hosted and internally sponsored by the Neurological Surgery Department of Oregon Health and Science Institute, which acts as a secure host for the confidential patient data of all participating centers around the world.

The Global NeuroSurg collaborative recognizes the importance of engaging medical students in Global Neurosurgery (1); therefore, we implement a novel collaborative research model that supports the involvement of medical students in global neurosurgery research.

This model is justified by the fact that the ratio of neurosurgeons to the population is low in many LMICs. Therefore, research collaboration in global neurosurgery that starts by inviting senior neurosurgeons to join and participate in the studies might lead to a low representation of low-resource settings where neurosurgeons are few, busy, or unreachable. Hence, this conventional research model is susceptible to selection bias where the resulting datasets will be skewed away from the low-resource settings.

To overcome this problem, the Global NeuroSurg research Collaborative uses medical students who are more available to provide access to such low-resource settings. In this model, medical students act as a contact point who provide access to the healthcare centers after gaining support from consultants, who act as local research leads for the project.

For example, in the Global NeuroSurg I (GNS-I) study on the management and outcomes of traumatic brain injury (TBI) in low-, middle-, and high-income countries (2), the study was initially delivered by (1) contacting neurosurgeons from the personal connections of the steering committee and (2) using the International Federation of Medical Students Associations (IFMSA). The National Officer of Medical Education in Egypt shared the study invitation with 2,000+ medical students from all countries around the world. Only after students have registered on the GNS-I study website, they are asked to identify a neurosurgeon, intensivist, or neurologist who has direct responsibility for TBI cases in their setting.

In the GNS-I study, teams can include up to three members per team, collecting data of consecutive TBI cases for 2 weeks; however, at least one member should be a neurosurgeon who provides care to TBI patients. Examples of some team structures with maximum student involvement are shown in Figure 1.

**Figure 1:**
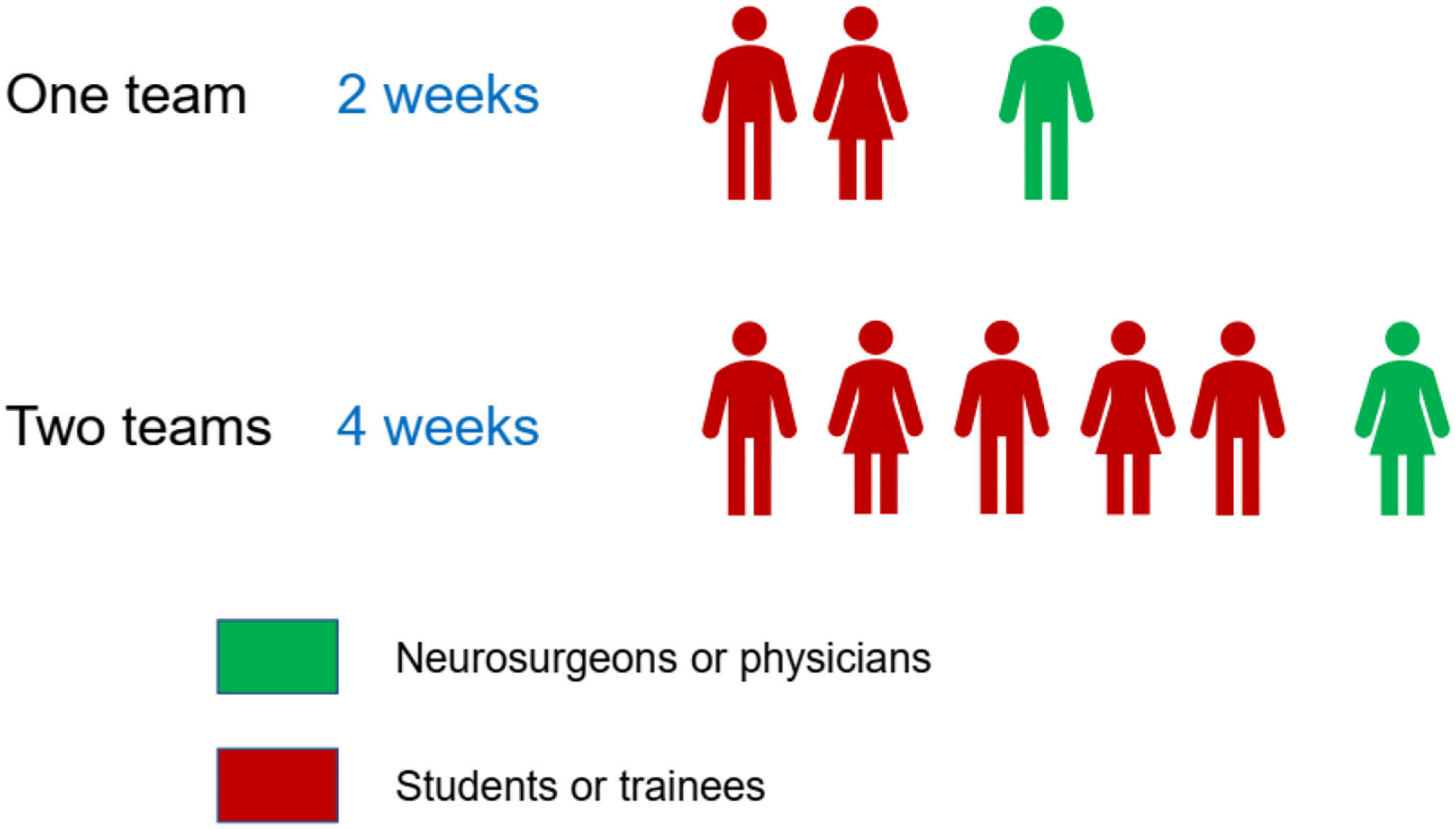
Examples for GNS-I study team structures with maximum student representation in the same center.

Teams can also include neurosurgeons only. However, we encourage student participation whenever possible. As of July 10, 2021, 2,030 collaborators from 84 countries have registered in the first study of the Global NeuroSurg research collaborative (Global NeurosurgI study). Of them, 1,550 are medical students (76% of all collaborators; Figure 2).

**Figure 2:**
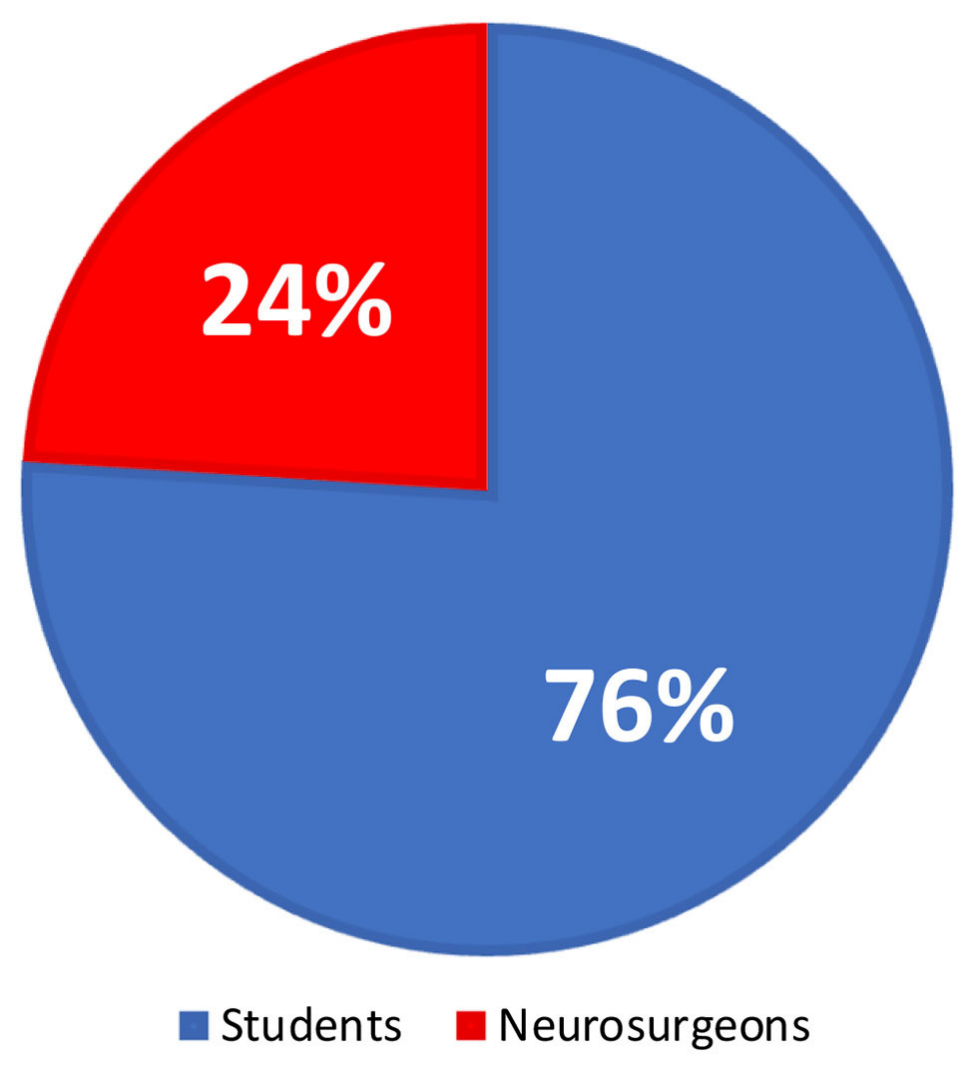
A pie chart of the representation of medical students among all the GNS-I study collaborators.

The model of Global NeuroSurg research collaborative relies on creating a grassroots movement among medical students worldwide, which increases the access and coverage of the global neurosurgical workforce. A geo map representing the 84 countries from which collaborators have registered for the GNS-I study is shown in Figure 3.

**Figure 3:**
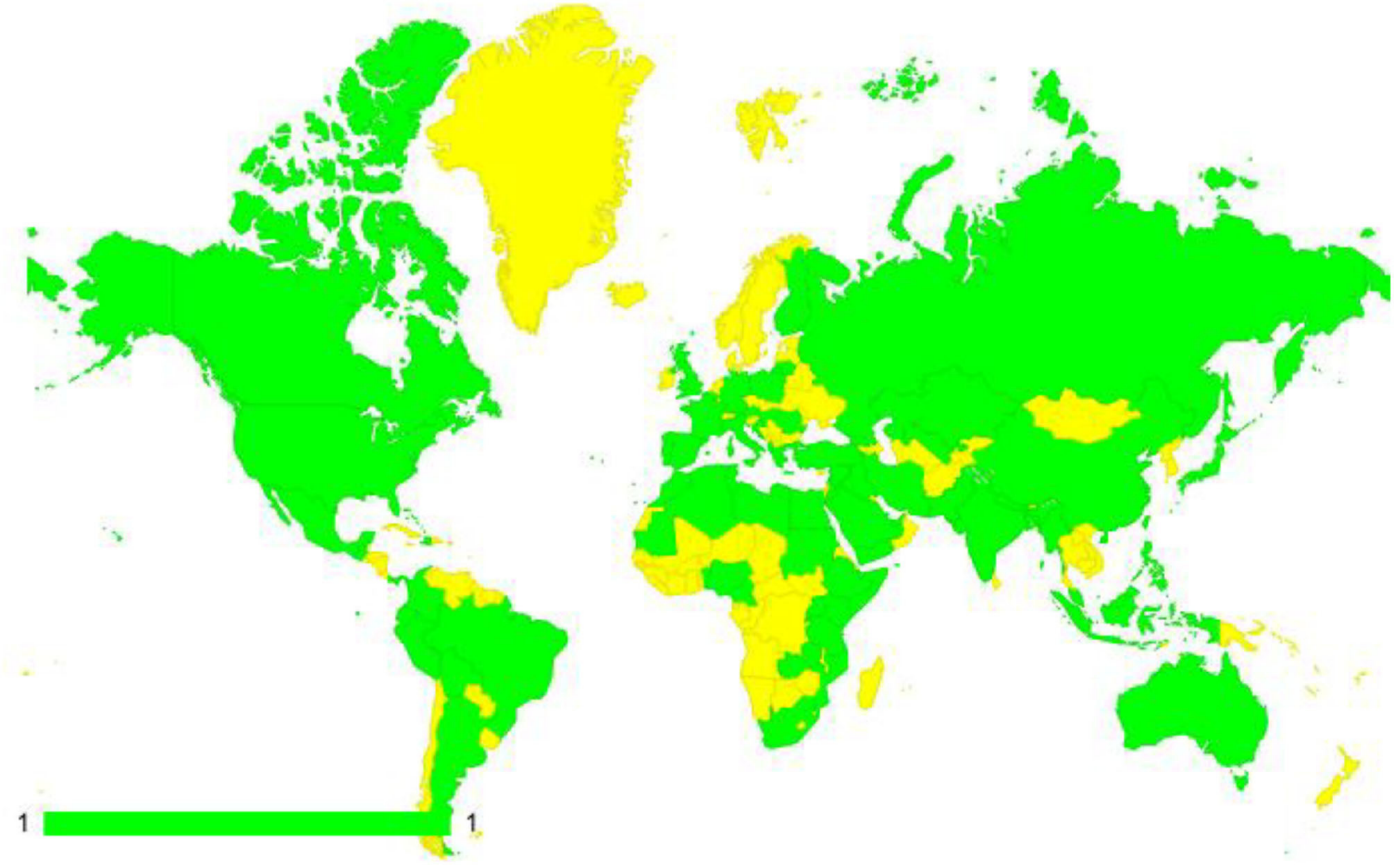
A geo map of the 84 countries from which collaborators have registered for the GNS-I study (marked in green color). The yellow color represents countries that are not yet registered.

Finally, we acknowledge that student- and trainee-led surgical research collaboratives are popular in the United Kingdom on the local and national levels as STARSurg collaborative and Surgical Trainees in East of England Research Collaborative Group; however, to the best of our knowledge, no collaborative groups have done the same in the global neurosurgery field. We believe that students are a proxy for low-resource settings and can help to expand the coverage of global neurosurgery research to include many underserved areas.

In the upcoming years, the Global NeuroSurg Research Collaborative will audit other areas of neurosurgical care around the world including stroke, epilepsy, hydrocephalus, and brain cancer surgeries. It is expected that these audit evaluations will identify the practices and factors associated with better patient outcomes and therefore, these future studies will inform neurosurgeons and decision makers. These studies will also highlight the gaps in neurosurgical care between HICs and LMICs, which will be a valuable tool for global neurosurgery advocacy, practice, and education.

In the future, it will be helpful to evaluate the benefits of involvement of medical students in the Global NeuroSurg research collaborative; however, we have not yet acquired the data to answer this question since the entire collaborative efforts are currently focused on finalizing a successful Global NeuroSurg I project. It is expected that the involvement of medical students in the Global NeuroSurg research collaborative will (1) increase their exposure to the neurosurgical OR, (2) enhance their participation in neurosurgical research and increase their CV and publication list, and (3) build their connections with the neurosurgical community.

## Author Contributions

All authors listed have made a substantial, direct and intellectual contribution to the work, and approved it for publication.

## Conflict of Interest

AN and AR are co-founders and leads of the Global NeuroSurg Research Collaborative.

## Publisher's Note

All claims expressed in this article are solely those of the authors and do not necessarily represent those of their affiliated organizations, or those of the publisher, the editors and the reviewers. Any product that may be evaluated in this article, or claim that may be made by its manufacturer, is not guaranteed or endorsed by the publisher.
